# Combining Regulatory T Cell Depletion and Inhibitory Receptor Blockade Improves Reactivation of Exhausted Virus-Specific CD8^+^ T Cells and Efficiently Reduces Chronic Retroviral Loads

**DOI:** 10.1371/journal.ppat.1003798

**Published:** 2013-12-05

**Authors:** Kirsten K. Dietze, Gennadiy Zelinskyy, Jia Liu, Freya Kretzmer, Simone Schimmer, Ulf Dittmer

**Affiliations:** Institute for Virology, University Hospital Essen, University of Duisburg-Essen, Essen, Germany; National Institute of Allergy and Infectious Diseases, National Institutes of Health, United States of America

## Abstract

Chronic infections with human viruses, such as HIV and HCV, or mouse viruses, such as LCMV or Friend Virus (FV), result in functional exhaustion of CD8^+^ T cells. Two main mechanisms have been described that mediate this exhaustion: expression of inhibitory receptors on CD8^+^ T cells and expansion of regulatory T cells (Tregs) that suppress CD8^+^ T cell activity. Several studies show that blockage of one of these pathways results in reactivation of CD8^+^ T cells and partial reduction in chronic viral loads. Using blocking antibodies against PD-1 ligand and Tim-3 and transgenic mice in which Tregs can be selectively ablated, we compared these two treatment strategies and combined them for the first time in a model of chronic retrovirus infection. Blocking inhibitory receptors was more efficient than transient depletion of Tregs in reactivating exhausted CD8^+^ T cells and reducing viral set points. However, a combination therapy was superior to any single treatment and further augmented CD8^+^ T cell responses and resulted in a sustained reduction in chronic viral loads. These results demonstrate that Tregs and inhibitory receptors are non-overlapping factors in the maintenance of chronic viral infections and that immunotherapies targeting both pathways may be a promising strategy to treat chronic infectious diseases.

## Author Summary

A loss of function, the so-called ‘exhaustion’ of CD8^+^ T cells, is a hallmark of many chronic infections. The T cell exhaustion is mediated by two main mechanisms, the expression of inhibitory receptors on CD8^+^ T cells and virus-induced expansion of regulatory T cells (Tregs), which suppress CD8^+^ T cell activity. Several mouse studies revealed a reactivation of CD8^+^ T cells and reduction in chronic viral loads after blockage of one of these pathways. These results initiated a number of clinical studies mainly with cancer patients, in which blocking antibodies were used to interfere with inhibitory receptor signaling or drugs that deplete Tregs. For the first time we combined the two therapeutic approaches by using transgenic mice in which Tregs can be selectively ablated and injection of blocking antibodies in a chronic retroviral infection. The results indicate that the combination therapy was superior to any single treatment in further augmenting CD8^+^ T cell responses and reducing chronic viral loads. Our findings demonstrate that Tregs and inhibitory receptors are non-overlapping factors in the maintenance of chronic viral infections and that immunotherapies targeting both pathways may be a promising new strategy to treat chronic infectious diseases.

## Introduction

Cytotoxic CD8^+^ T cells are crucial for the control of most virus infections. However, in several chronic virus infections, like HIV or hepatitis C virus (HCV) in humans, the virus evades destruction by CD8^+^ T cells. Mostly these infections are associated with an appearance of functionally exhausted virus-specific effector cells, which reflects an important mechanism of immune evasion and likely contributes to the inability of the host to eliminate the pathogen. There are two main mechanisms described in the context of functional disability of CD8^+^ T cells. One of these mechanisms appears to be the induction of Tregs, a specialized CD4- and Foxp3-expressing T cell subset that controls immune responses by suppressing the proliferation and functions of effector T cells. The mechanism of viral immune escape by induction of Tregs was first described in studies using the Friend retrovirus (FV) infection of mice [1]. We demonstrated that acute FV infection induces expansion of two distinct Treg subpopulations [2]. The expansion was partly dependent on the magnitude of the virus-specific CD8^+^ T cell response. In turn the Tregs negatively influenced the peak CD8^+^ T cell response contributing to the establishment and maintenance of long-term chronic FV infections [2], [3]. The depletion of Tregs during the acute phase of infection resulted in enhanced effector T cell function and decreased viral loads [3], [4]. In an established chronic infection the Treg pool is reduced compared to its peak expansion after acute infection, but still significantly enlarged as compared to the pool of naive mice (data not shown and [3]). A transient depletion of Tregs in an established chronic infection improved anti-viral immune responses in part by reactivating previously suppressed and functionally exhausted CD8^+^ T cells and thereby significantly reduced chronic viral set points [5].

Another important mechanism associated with the appearance of dysfunctional CD8^+^ T cells is the signaling of inhibitory receptors, which induces CD8^+^ T cell exhaustion. One of the prototypic inhibitory receptors described as an important mediator of T cell exhaustion in chronic viral infections is programmed death-1 (PD-1). The PD-1 receptor is a negative regulator of T cell proliferation and activation and is known to mediate suppressive functions after binding to its ligands PD-L1 or PD-L2 [6], [7]. Another important inhibitory receptor known to play a role in CD8^+^ T cell dysfunction is T cell immunoglobulin and mucin domain 3 (Tim-3), which is thought to recognize the ligand galectin-9 (galactose-specific soluble lectin 9) [8]. Increased levels of these inhibitory receptors were found on the surface of exhausted CD8^+^ T cells from patients chronically infected with HCV, HIV or hepatitis B virus (HBV) [9], [10], [11]. In addition, high levels of these inhibitory receptors were also found on CD8^+^ T cells from mice chronically infected with LCMV or FV [12], [13] as well as on CD8^+^ T cells from rhesus macaques chronically infected with simian immunodeficiency virus (SIV) [14]. In several mouse and monkey studies a temporary blockade of the interactions between these receptors and their ligands resulted in a partial reconstitution of effector functions of exhausted virus-specific CD8^+^ T cells and reduced viral loads during chronic infections [6], [7], [12], [14], [15]. Additionally, a successful clinical trial of PD-1–blocking was recently completed in patients with different types of cancer [16]. Despite these encouraging results the current studies indicate that blocking inhibitory receptors or manipulating Tregs only partially reactivates CD8^+^ T cells and do not completely eliminate chronic virus. We therefore decided to combine these two therapeutic approaches, which until now has only been done in a study investigating the pathogenesis of acute murine AIDS [17], but never been performed in an established chronic virus infection. As a model we choose the chronic infection of mice with FV, a life-long persistent infection that is characterized by constant low-level virus replication and profound functional exhaustion of CD8^+^ T cells [18].

## Results and Discussion

**Figure 1:**
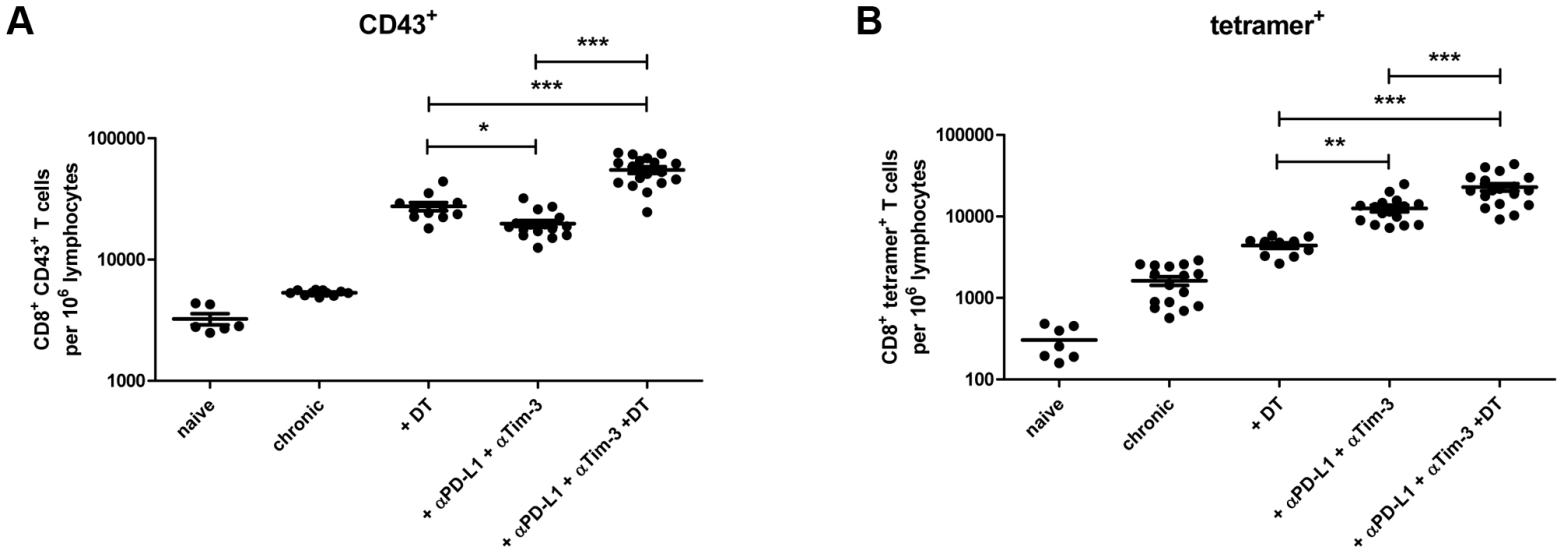
CD8^+^ T cell functions after Treg depletion and/or inhibitory pathway blockage in chronically infected mice. Mice chronically infected with FV were treated with DT (DEREG mice) and/or blocking antibodies against PD-L1 and TIM-3 as indicated. Frequencies of (**A**) total activated (expressing the activation-induced isoform of CD43) CD8^+^ T cells and (**B**) virus-specific class I tetramer^+^ CD8^+^ T cells are shown as calculated by flow cytometry. Each dot represents an individual mouse. Data were pooled from 3 to 5 independent experiments with similar results. Statistically significant differences are indicated by asterisks (*<0.05; **<0.005; ***<0.0005; analysis of variance [ANOVA], Newman-Keuls multiple comparison test).

**Figure 2:**
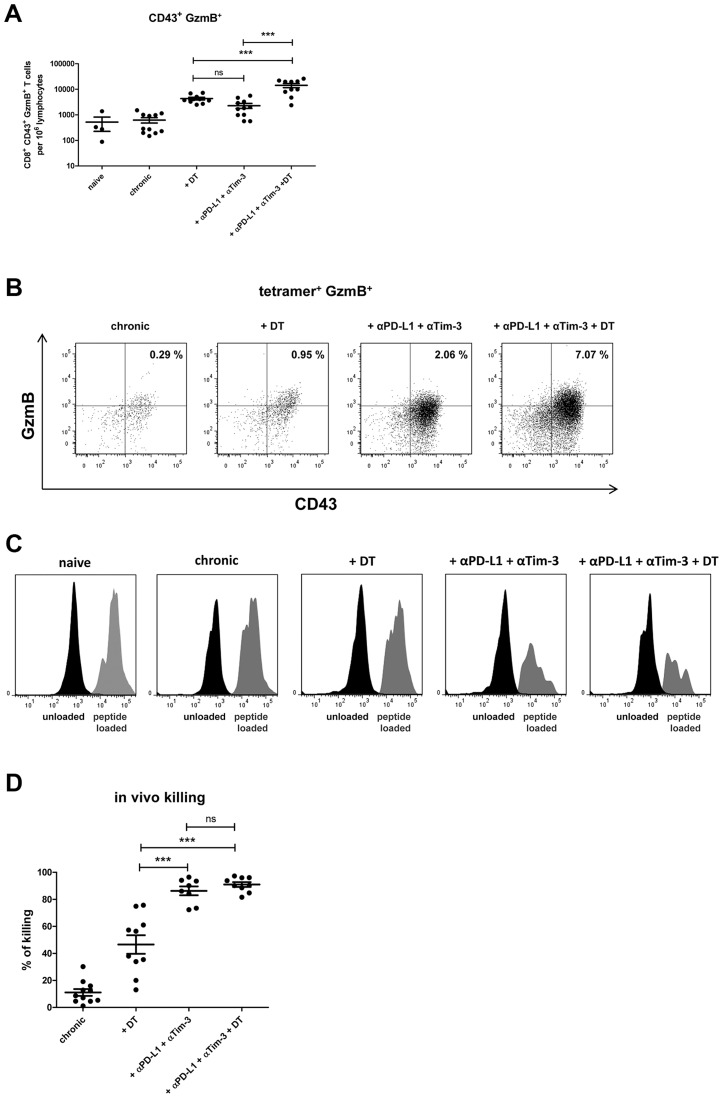
CD8^+^ T cell activity in chronically infected mice after Treg depletion and/or blocking of inhibitory pathways. Mice chronically infected with FV were treated with DT (DEREG mice) and/or blocking antibodies against PD-L1 and TIM-3 as indicated. (**A**) Frequencies of CD8^+^ CD43^+^ granzyme B^+^ (GzmB) T cells are shown as calculated by flow cytometry. Each dot represents an individual mouse. Data were pooled from 3 to 5 independent experiments with similar results. Statistically significant differences are indicated by asterisks (*<0.05; **<0.005; ***<0.0005; analysis of variance [ANOVA], Newman-Keuls multiple comparison test). (**B**) Representative dot plots for GzmB production in virus-specific (tetramer^+^) CD8^+^ T cells. The percentages of tetramer^+^ CD8^+^ T cells that were CD43^+^ and expressed GzmB are given in the upper right quadrants. (**C**) Representative histograms showing differential killing of unloaded cells (CFSE^low^, black) versus target cells loaded with the FV D^b^-GagL peptide (CFSE^hi^, grey) in spleens using an *in vivo* cytotoxicity assay. (**D**) Percentages of target cell killing in spleens of mice from the differently treated groups. Data were pooled from spleen cells of 8–11 mice per group from 2 independent experiments. Statistically significant differences are indicated by asterisks (***<0.0005; analysis of variance [ANOVA], Newman-Keuls multiple comparison test).

DEREG mice [19] were infected with FV and rested for >60 days to establish chronic infection. During chronic FV infection specific CD8^+^ T cells are present with an exhausted phenotype [20] and expressed high levels of the inhibitory receptors PD-1 and Tim-3 (Fig. S2). In addition, they have expanded populations of activated, but non-virus-specific Tregs in lymphoid tissues [3], [21], [22]. The viral set points are low (mean of about 10 infected cells per 10^6^ spleen cells) and relatively consistent between individual animals. After 60 days of infection, one group of mice received diphtheria toxin (DT), which resulted in over 97% depletion of eGFP^+^ Tregs [3], [4]. Another group of animals was injected with monoclonal antibodies against PD-L1 and Tim-3 to block inhibitory receptor signaling on CD8^+^ T cells. After depletion of Tregs a more than 5-fold increase in the mean frequency of total activated (CD43^+^) CD8^+^ T cells was detected in the spleen of chronically infected mice (Fig. 1A). A 4-fold expansion of CD43^+^ CD8^+^ T cells was also found in mice treated with α-PD-L1 and α-TIM-3 but it was significantly lower than in the group of Treg depleted mice. Activated CD43^+^ CD8^+^ T cells also had increased expression of CD44 and were negative for CD62L demonstrating their effector phenotype (data not shown and [22]). Tetramers specific for the immunodominant FV epitope D^b^-GagL [23] were then used to determine augmentation of virus-specific CD8^+^ T cell responses. Treg ablation resulted in a 2.5-fold increase in the mean frequency of tetramer positive CD8^+^ T cells compared to non-depleted controls (Fig. 1B). Interestingly, in this assay the increased frequencies in FV-specific T cells was significantly higher in the group of mice receiving the blocking antibodies (7-fold) than those receiving DT. Hence, Treg depletion resulted in a higher expansion of the total population of activated CD8^+^ T cells compared to inhibitory receptor blockage, whereas it was the other way around for the expansion of the tetramer^+^ cells recognizing the immunodominant FV CD8^+^ T cell epitope (Fig. 1A and B). From studies with LCMV it is known that CD8^+^ T cells specific for immunodominant epitopes tend to expand more efficiently than the ones recognizing subdominant epitopes after treatment with blocking antibodies [6], [24]. In contrast, Treg depletion most likely affects all activated T cells rather equally. Thus, blocking inhibitory receptors seems to be most effective in reactivating terminally differentiated T cells because those are the ones that express the highest levels of inhibitory receptors [13], whereas Treg depletion may be more efficient to reactivate the total CD8^+^ T cell effector population. Since virus-specific CD8^+^ T cells are exhausted during chronic FV infection [20], we determined the level of functional reactivation after Treg depletion or inhibitory receptor signaling blockade. The cytolytic capacity of CD8^+^ T cells is a key factor in FV control [25], so we focused on analyzing the production of the cytotoxic molecule granzyme B and the in vivo killing activity of CD8^+^ T cells after treatment. Activated CD8^+^ T cells from chronically infected mice expressed very low levels of granzyme B, which were indistinguishable from those of naive mice. Consequently, no killing of splenocytes loaded with the FV immunodominant epitope [3], [23] was found in an in vivo CTL assay. Following Treg depletion as well as receptor blockade significantly more of the total activated CD8^+^ T cells expressed granzyme B (Fig. 2A). This was also the case for the subset of tetramer^+^ CD8^+^ T cells but for these cells recognizing the immunodominant FV epitope the treatment with α-PD-L1 and α-TIM-3 was more efficient for granzyme B induction than Treg depletion (Fig. 2B). Similar findings were made in the in vivo CTL assay. Treg-depleted mice averaged 46% killing of peptide-loaded targets whereas the killing activity was with almost 86% significantly higher in chronically infected mice receiving the blocking antibodies (Fig. 2C and D). These data demonstrate that reactivation of cytotoxic CD8^+^ T cell function in chronic retroviral infection is more pronounced when inhibitory receptors on CD8^+^ T cells were blocked compared to depletion of Tregs.

**Figure 3:**
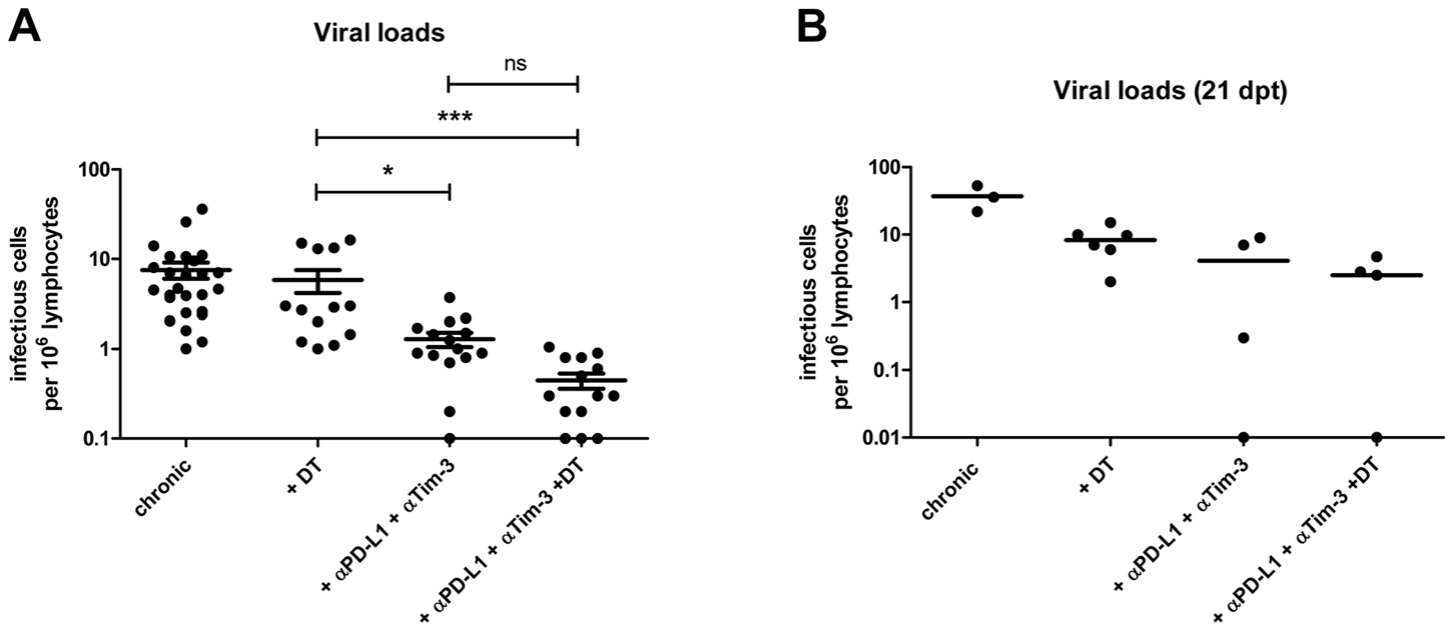
Viral loads in chronic infection after Treg depletion and/or blocking of inhibitory pathways. (**A**) Spleens of chronically FV-infected mice from the different treatment groups were analyzed for viral loads by infectious center assays 1 day after termination of treatment. Each dot represents an individual mouse. Data were pooled from 4 to 6 independent experiments with similar results. Statistically significant differences are indicated by asterisks (*<0.05; ***<0.0005; analysis of variance [ANOVA] nonparametric, Dunn's multiple comparison test). (**B**) To study long-term effects of the treatment spleens of mice from the different groups were analyzed for viral loads by infectious center assay at 21 days post treatment (dpt). Each dot represents an individual mouse.

This suggested that α-PD-L1 and α-TIM-3 treatment might be more potent than ablation of Tregs in diminishing chronic virus loads. Indeed, we found a significant difference in viral loads between these two groups one day post treatment (Fig. 3A) indicating that the strongly enhanced cytotoxic activity of FV-specific CD8^+^ T cells after blockage of inhibitory receptors correlated with superior virus control. As previously published the therapeutic effect of Treg depletion was sustained [5] with reduced viral loads still detectable at 21 days post treatment (Fig. 3B). In addition, the viral set points remained also reduced 21 days after cessation of antibody blockage (Fig. 3B).

As a next step we performed a combination therapy with DT injection plus antibody treatment in chronically infected mice. The results demonstrated an additive therapeutic effect with significant higher frequencies of total activated (CD43^+^) and tetramer^+^ CD8^+^ T cells than after any of the two single treatments (Fig. 1A and B). Further characterization of the CD8^+^ T cells revealed that the combination therapy induced proliferation (Fig. S3A) and increased IFN-γ production by activated CD8^+^ T cells (Fig. S3B and C). Virus-specific CD8^+^ T cells were high in KLRG1 (Killer cell lectin-like receptor G1) expression after combination therapy confirming their terminally differentiated effector phenotype (Fig. S3D). Granzyme B expression was also further enhanced in total activated CD8^+^ T cells (Fig. 2A) as well as in virus-specific CD8^+^ T cells (Fig. 2B) after combination therapy. However, this was only reflected in augmented FV-specific target cell killing when Treg depleted mice were compared to the animals receiving DT plus α-PD-L1 and α-TIM-3. No significant difference was found between mice treated with antibodies plus DT and antibodies alone, most likely because the target cell killing in this group was already at a mean of 86% (Fig. 2D). The significantly enhanced CD8^+^ T cell killing resulted in significantly reduced chronic viral loads when Treg ablated mice were compared to the mouse group receiving combination therapy (Fig. 3A). The combined therapy also reduced the viral set points in comparison to the group of mice in which only the inhibitory receptors were blocked, but this difference was not significant using the one-way ANOVA and Newman-Keuls multiple comparison test. However, when only the α-PD-L1 and α-TIM-3 versus the α-PD-L1 and α-TIM-3 plus DT group were compared with a Student's *t*-test the reduction was highly significant (p = 0.0009) (Fig. 3A). Furthermore, an analysis performed three weeks after termination of treatment demonstrated a sustained reduction in chronic viral loads after combination therapy (Fig. 3B). At that time, CD8^+^ T cells still possessed slightly enhanced functional properties such as higher activation levels (CD62L^−^ CD43^+^) and increased IFN-γ production, although the Treg compartment was fully restored 21 days after the last DT injection (data not shown).

The current experiments show that both Tregs and inhibitory receptors are independent factors mediating T cell exhaustion during chronic infection. Until now the exact molecular mechanism of CD8^+^ T cell suppression by Tregs has not been unraveled for most chronic viral infections, but studies suggest that it is cell-to-cell contact dependent [26], [27]. Since ligands for inhibitory receptors were found to be highly expressed on Tregs [28] these cells might be able to inhibit effector T cells by direct interaction of their ligands with inhibitory receptors. In this scenario, depleting Tregs and blocking inhibitory signals with antibodies would target the same suppressive mechanism. However, our current data show that Tregs and inhibitory receptors are mostly non-overlapping mechanisms of T cell exhaustion and that blocking both pathways has an additive therapeutic effect during chronic retroviral infection. Another cell-to-cell contact dependent mechanism of Treg suppression, like the cyclic AMP (Adenosine monophosphate) that has been described in HIV infection [27], are therefore more likely contributing to functional T cell exhaustion.

Our findings have obvious implications for therapeutic strategies to treat chronic infections. A reduction of chronic viral loads following a temporary treatment could be beneficial for many chronic viral infections in humans. This is especially true for HIV infection where chronic viral set points strongly influence disease progression [29]. Similar to chronic FV infection, Tregs accumulate in lymphoid tissues of HIV-infected patients [30], [31] and the effector T cells upregulate inhibitory receptors [32], which correlates with CD8^+^ T cell exhaustion and local viral loads [33]. Similar findings were made in studies with HCV-infected individuals [34], [35], [36], suggesting that these patients could benefit from a combination therapy of Treg manipulation and inhibitory receptor blockade. One obvious concern with Treg manipulation is that this may result in immunopathology or autoimmunity. However, in the current and in previous experiments transient ablation of Tregs in mice infected with FV did not result in any clinical symptoms of immunopathology ([3] and data not shown). Furthermore, clinical trials with melanoma patients in which a recombinant IL-2-diphteria toxin fusion protein (ONTAK®) was administered [37], demonstrated that temporary Treg depletion did not induce serious clinical side effects in humans.

These results demonstrate that Tregs and inhibitory receptors may both be targeted at the same time in new combination therapies as a promising strategy to treat chronic infectious diseases.

## Methods

### Ethics statement

Animal experiments were performed in strict accordance with the German regulations of the Society for Laboratory Animal Science (GV-SOLAS) and the European Health Law of the Federation of Laboratory Animal Science Associations (FELASA). The protocol was approved by the North Rhine-Westphalia State Agency for Nature, Environment and Consumer Protection (LANUV) (Permit number: G 1208/11 and G 1341/12). All efforts were made to minimize suffering.

#### Mice

Inbred C57BL/6 (B6) and DEREG [19] mice were maintained under pathogen free conditions. Experiments were done using mice (H-2^b/b^, Fv1^b/b^, Fv2^r/r^) or transgenic mice backcrossed on C57BL/6 background that are resistant to FV-induced leukemia. All mice were females of 8–12 weeks of age at the beginning of the experiments.

### Virus and viral infection

The FV stock used in these experiment was FV complex containing B-tropic Friend murine leukemia helper virus (F-MuLV) and polycythemia-inducing spleen focus-forming virus [38]. The stock was prepared as a 10% spleen cell homogenate from BALB/c mice infected 14 days previously with 3,000 spleen focus-forming units of non-cloned virus stock. Experimental mice were injected intravenously with 60,000 spleen focus-forming units of FV complex and additional 2×10^5^ units of F-MuLV to enhance chronic FV loads.

The virus stock did not contain lactate dehydrogenase-elevating virus.

### Infectious center assays

Infectious center assays were performed as described previously [39].

### Cell surface and intracellular staining by flow cytometry

Surface and intracellular staining were performed as described previously [5]. Data were acquired on a LSR II flow cytometer (Becton Dickinson) from 350,000–500,000 lymphocyte-gated events per sample. Analyses were done using FACSDiva software (Becton Dickinson) and FlowJo software (Treestar).

### Lymphocyte depletion

To deplete Tregs, chronically FV-infected DEREG mice were injected intraperitoneally with diphtheria toxin (Merck, Darmstadt, Germany), diluted in endotoxin-free PBS. 0.5 µg DT was inoculated every third day for 3 times. The treatment depleted over 97% of the CD4^+^ eGFP^+^ T cells in all investigated organs of DEREG mice. The T cell responses and viral loads were analyzed 1 or 21 days post treatment.

### *In vivo* blockade

For blockade of the PD-1 pathway in chronically FV-infected mice, 200 µg rat anti-mouse PD-L1 Ab (10F.9G2; BioXCell) was administered intraperitoneally every third day for 4 times. To block the Tim-3 pathway, 100 µg rat anti-mouse Tim-3 Ab (RMT3-23; BioXCell) was administered intraperitoneally every other day for 4 times. The anti-PD-L1 treatment did not influence the PD-1 expression on CD8^+^ T cells from chronically infected mice, whereas the direct blocking of the Tim-3 receptor could be nicely demonstrated by flow cytometry staining (Fig. S2). The T cell responses and viral loads were analyzed one day post treatments or 21 days post treatments (Fig. S1)

### Tetramers and tetramer staining

For detection of D^b^-GagL-specific CD8^+^ T cells, nucleated spleen cells were stained with PE labeled MHC class I H2-D^b^ tetramers specific for FV GagL peptide [23], [40] (Beckman Coulter, Marseille, France).

### *In vivo* cytotoxicity assay

The *in vivo* CTL assay described by Barber et al. [41] was modified to measure cytotoxicity in FV-infected mice [3].

### Statistical analysis

Statistical data were derived by using the GraphPad Prism software (GraphPad Software). Data were analyzed using one-way ANOVA and Newman-Keuls multiple comparison test, non-parametric one-way ANOVA and Dunn's multiple comparison test or Student's *t*-test.

## Supporting Information

Figure S1 **Treatment protocol.** Chronically infected mice were treated with DT and/or blocking Abs as shown in the diagram. Treg depletion: chronically FV-infected DEREG mice were administered with DT every third day for 3 times. Blocking Abs: chronically FV-infected mice were administered with antibodies against PD-L1 every third day for 4 times and with antibodies against Tim-3 every other day for 4 times. Anti-PD-L1 treatment was started three days earlier than the anti-TIM-3 or DT infection. Combination therapy: chronically FV-infected DEREG mice were administered with DT every third day for 3 times, with antibodies against PD-L1 every third day for 4 times and with antibodies against Tim-3 every other day for 4 times. Mice were analyzed either 1 or 21 days post treatment.
(TIF)

Figure S2 **PD-1 and Tim-3 expression on virus-specific (tetramer^+^) CD8^+^ T cells.** Representative histograms of differential PD-1 and Tim-3 expression on CD8^+^ T cells from spleens of naive mice (grey area) and on virus-specific (tetramer^+^) CD8^+^ T cells from spleens of chronically FV-infected mice (black lines). The different experimental groups are indicated on the right.
(TIF)

Figure S3 **Characteristics of CD8^+^ T cells in chronically infected mice after Treg depletion and blocking of inhibitory pathways.** DEREG mice chronically infected with FV were treated with DT and blocking antibodies against PD-L1 and TIM-3 as indicated. Frequencies of (**A**) proliferating Ki-67^+^ CD8^+^ T cells and (**B**) IFN-γ-producing CD8^+^ CD43^+^ T cells are shown as calculated by flow cytometry. Each column represents the mean frequency plus SEM for a group of 3–5 mice. (**C**) Representative dot plots for IFN-γ production in CD8^+^ T cells. The percentages of CD8^+^ T cells that were CD43^+^ and produced IFN-γ are given in the upper right quadrants. (**D**) Frequencies of terminal differentiated (CD127^−^ KLRG1^+^) virus-specific (tetramer^+^) effector CD8^+^ T cells are shown as calculated by flow cytometry. Each column represents the mean frequency plus SEM for a group of 3–5 mice.
(TIF)
