# Supplementary figures and images for: Combining Regulatory T Cell Depletion and Inhibitory Receptor Blockade Improves Reactivation of Exhausted Virus-Specific CD8+ T Cells and Efficiently Reduces Chronic Retroviral Loads
Source: PLoS Pathog. 2013 Dec 5;9(12):e1003798. doi: 10.1371/journal.ppat.1003798 (PMC3855586; doi:10.1371/journal.ppat.1003798)

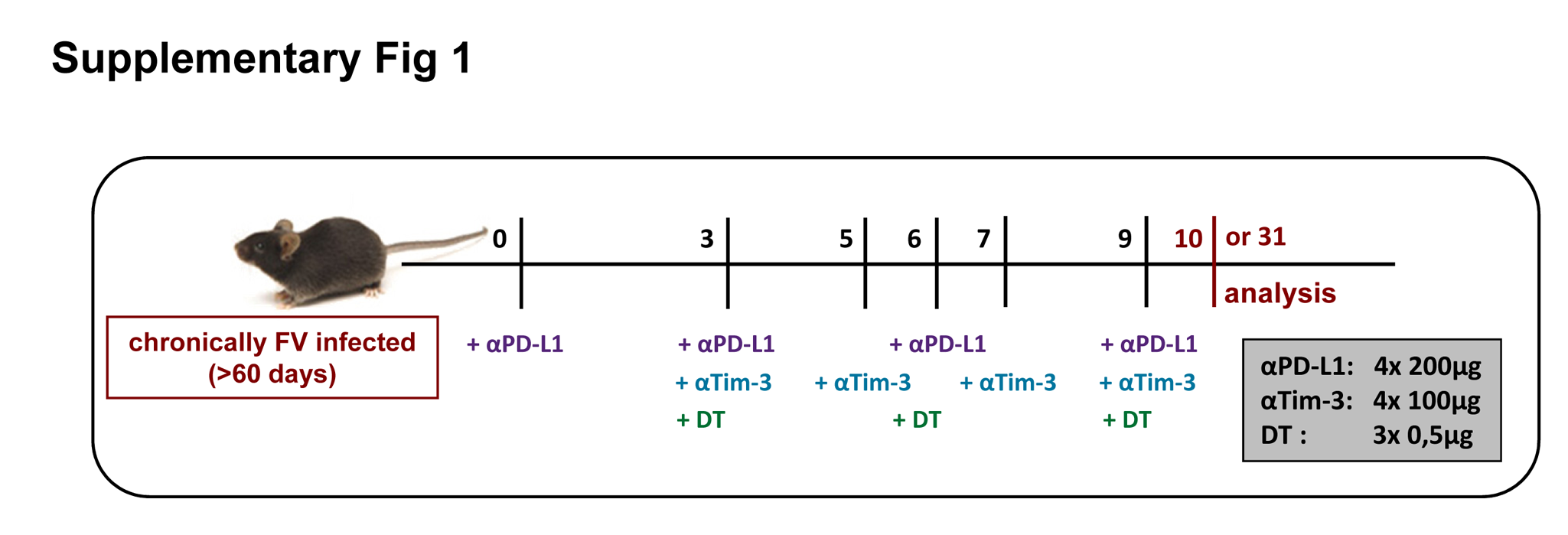

Supplement: Figure S1 — Treatment protocol. Chronically infected mice were treated with DT and/or blocking Abs as shown in the diagram. Treg depletion: chronically FV-infected DEREG mice were administered with DT every third day for 3 times. Blocking Abs: chronically FV-infected mice were administered with antibodies against PD-L1 every third day for 4 times and with antibodies against Tim-3 every other day for 4 times. Anti-PD-L1 treatment was started three days earlier than the anti-TIM-3 or DT infection. Combination therapy: chronically FV-infected DEREG mice were administered with DT every third day for 3 times, with antibodies against PD-L1 every third day for 4 times and with antibodies against Tim-3 every other day for 4 times. Mice were analyzed either 1 or 21 days post treatment. (TIF) [file ppat.1003798.s001.tif]

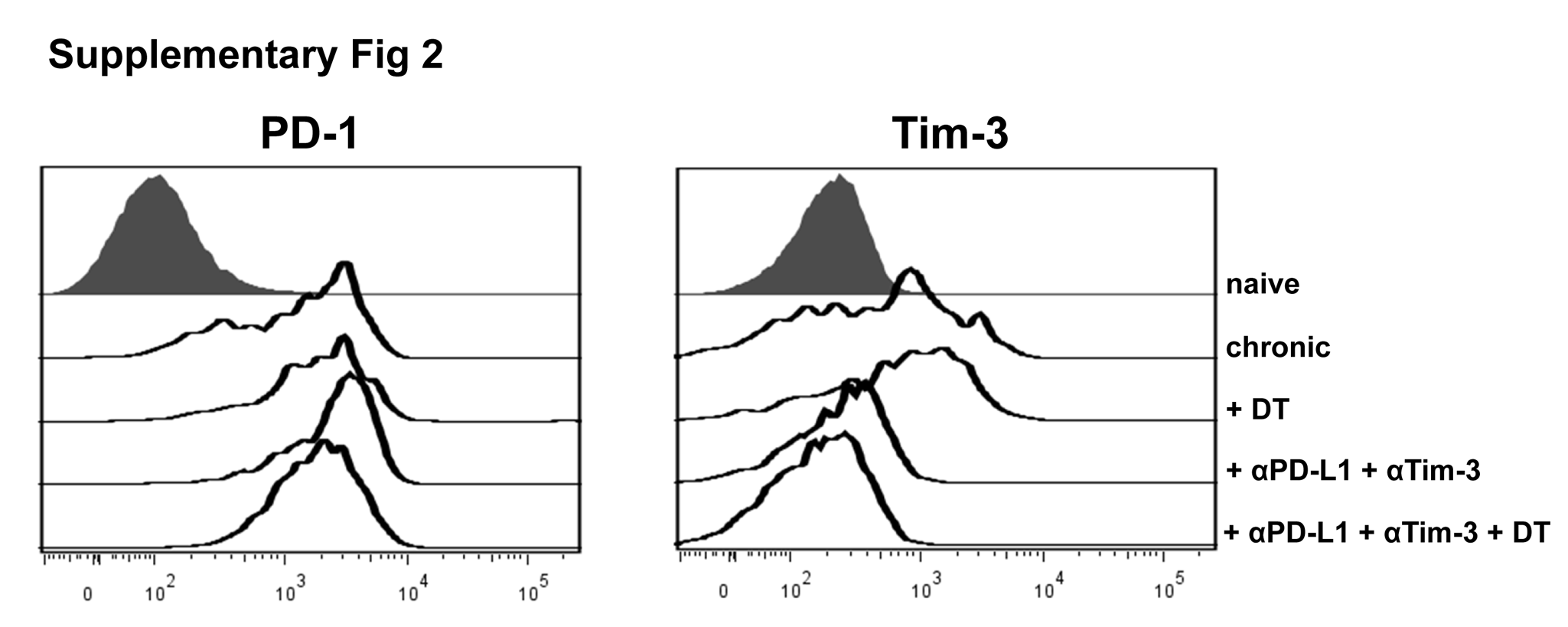

Supplement: Figure S2 — PD-1 and Tim-3 expression on virus-specific (tetramer+) CD8+ T cells. Representative histograms of differential PD-1 and Tim-3 expression on CD8+ T cells from spleens of naive mice (grey area) and on virus-specific (tetramer+) CD8+ T cells from spleens of chronically FV-infected mice (black lines). The different experimental groups are indicated on the right. (TIF) [file ppat.1003798.s002.tif]

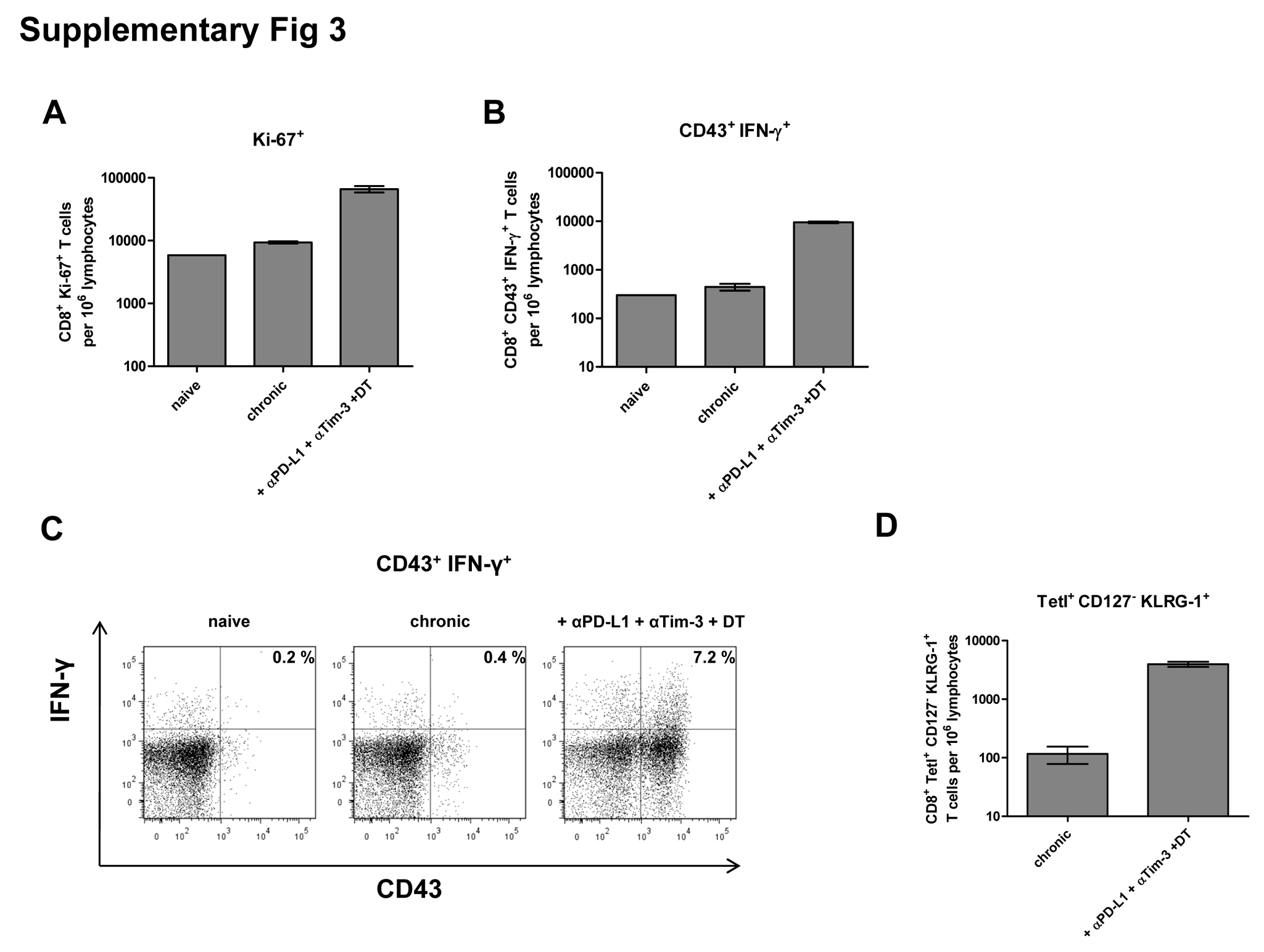

Supplement: Figure S3 — Characteristics of CD8+ T cells in chronically infected mice after Treg depletion and blocking of inhibitory pathways. DEREG mice chronically infected with FV were treated with DT and blocking antibodies against PD-L1 and TIM-3 as indicated. Frequencies of (A) proliferating Ki-67+ CD8+ T cells and (B) IFN-γ-producing CD8+ CD43+ T cells are shown as calculated by flow cytometry. Each column represents the mean frequency plus SEM for a group of 3–5 mice. (C) Representative dot plots for IFN-γ production in CD8+ T cells. The percentages of CD8+ T cells that were CD43+ and produced IFN-γ are given in the upper right quadrants. (D) Frequencies of terminal differentiated (CD127− KLRG1+) virus-specific (tetramer+) effector CD8+ T cells are shown as calculated by flow cytometry. Each column represents the mean frequency plus SEM for a group of 3–5 mice. (TIF) [file ppat.1003798.s003.tif]
